# Compressive Strength-Based Classification of Eco-Friendly Concretes Using Machine Learning Models

**DOI:** 10.3390/ma18235344

**Published:** 2025-11-27

**Authors:** Daniel Alcala-Gonzalez, Luis F. Mateo, M. Ángeles Quijano, M. Isabel Más-López, Eva M. García-del-Toro

**Affiliations:** 1 Departamento de Ingeniería Civil: Hidráulica, Energía y Medio Ambiente, ETSI Caminos, Canales y Puertos-Edificio Retiro, Universidad Politécnica de Madrid, Alfonso XII, 3, 28014 Madrid, Spain; d.alcalag@upm.es (D.A.-G.); marian.quijano@upm.es (M.Á.Q.); 2 Departamento de Matemática e Informática Aplicadas a las Ingenierías Civil y Naval, ETSI Caminos, Canales y Puertos-Edificio Retiro, Universidad Politécnica de Madrid, Alfonso XII, 3, 28014 Madrid, Spain; luis.f.mateo@upm.es (L.F.M.); mariaisabel.mas@upm.es (M.I.M.-L.); 3 Centro I+D+i en Infraestructuras Civiles Inteligentes y Sostenibles (CIVILIS), ETSI Caminos, Canales y Puertos-Edificio Retiro, Universidad Politécnica de Madrid, Alfonso XII, 3, 28014 Madrid, Spain

**Keywords:** eco-friendly concrete, compressive strength, machine learning, Naïve Bayes, Random Forest, glass powder, sustainable construction, non-destructive evaluation

## Abstract

Accurate prediction of compressive strength in eco-friendly concretes, where part of the cement is replaced with recycled glass powder, remains a fundamental challenge for sustainable construction. This study evaluates and compares the performance of five machine learning models—Naïve Bayes, Random Forest, Decision Tree, Support Vector Machine (SVM), and k-Nearest Neighbors (k-NN)—for classifying the compressive strength of concretes with different mix designs and curing ages. The dataset includes 846 experimental samples produced at the School of Civil Engineering of UPM between 2004 and 2019. The results showed that Naïve Bayes and Random Forest achieved the highest accuracy and generalizability, confirming that the incorporation of glass powder does not introduce significant data instability and can serve as a viable and sustainable substitute of cement. The Decision Tree model provided the greatest interpretability, enabling insight into the influence of mixture parameters, while SVM and k-NN were primarily effective in extreme strength categories. Overall, the findings demonstrated that probabilistic and ensemble learning methods outperform deterministic and proximity-based algorithms in classifying materials with high compositional variability. This work reinforces the potential of artificial intelligence as a non-destructive, reliable, and scalable tool for optimizing the performance of low carbon concretes and promoting sustainable materials engineering.

## 1. Introduction

Concrete is the most widely used man-made material in civil engineering, with a significant impact on both the economy and the environment [1]. The construction sector contributes substantially to economic development (approximately 9% of the European Union’s GDP), generates millions of direct and indirect jobs (around 18 million in the EU), and fulfills the infrastructure and building needs of the population [2,3]. However, it is also one of the main consumers of natural resources, accounting for nearly 50% of total raw material use and 36% of global final energy consumption [4,5], making it a major source of greenhouse gas emissions [4,6].

In response, the scientific and technical community has focused on developing strategies aimed at reducing the consumption of non-renewable resources and emissions associated with concrete production, to promote a more sustainable and environmentally responsible industry [7,8]. Among the most promising alternatives are the recycling of material and the incorporation of mineral additives that partially replace traditional Portland cement, a material with high environmental impact due to emissions generated during its production [9,10,11]. In this context, eco-friendly concrete emerges as an innovative solution, where a fraction of cement is replaced with recycled glass powder derived from glass waste that, due to its physical or chemical properties, cannot be reintroduced into conventional recycling processes and would otherwise end up in landfills. This approach valorizes a difficult-to-manage byproduct, reduces the need for disposal, and actively contributes to circular economy principles and the mitigation of the sector’s environmental impact. In civil engineering, structures are designed to meet specific requirements based on their intended function, expected service life, and exposure conditions [12]. Within the design process, compressive strength is one of the most relevant properties, as it determines the quality and mechanical performance of concrete [13]. The type of concrete is selected according to its strength class (non-structural, ordinary structural, high strength, or very high-strength) based on project requirements and exposure conditions [14]. This classification is based on criteria established in international standards such as Eurocode 2 [15], ACI 318 [16], ACI 239 for UHPC [17], and the Spanish Structural Code [18], which specify minimum required strength levels and applicable conditions for each category. The experimental determination of concrete compressive strength is technically complex due to the nonlinear relationship between the composition of the constituent material and resulting mechanical behavior [19,20,21]. Traditionally, this involves casting multiple specimens cured under controlled conditions over periods ranging from 1 to 365 days [22], followed by destructive laboratory testing to determine compressive strength, which entails a significant cost in terms of time, materials, and labor. In this context, artificial intelligence (AI) and machine learning (ML) models offer a non-destructive and efficient approach for predicting and classifying compressive strength, particularly in eco-friendly concretes with partial cement replacement using non-recyclable glass powder. This represents a significant step toward digitalization and sustainability in the construction sector.

Over the past three decades, artificial neural networks (ANNs) have been successfully applied across a wide range of scientific and engineering disciplines, demonstrating remarkable capabilities for modeling complex phenomena and predicting non-linear behaviors [23,24,25]. Although their adoption in civil engineering has been relatively limited, several studies have shown their potential for estimating the compressive strength of concrete with accuracy comparable to or exceeding that of traditional empirical methods [26,27,28,29]. The quality of the predictions depends heavily on the network design, training parameters, and selection of input variables, enabling continuous optimization of the models and their adaptation to the specific requirements of each application. Beyond ANNs, a wide variety of AI techniques have been applied to concrete behavior studies, including Adaptive Neuro-Fuzzy Inference Systems (ANFIS), Genetic Programming (GP), Support Vector Machines (SVM), Classification and Regression Trees (CART), and Biogeography-Based Programming (BBP), among others [30]. These tools broaden the methodological spectrum for predictive modeling and mix optimization, reducing reliance on exhaustive experimental testing. Recent studies have proposed increasingly sophisticated approaches combining ML models with metaheuristic optimization algorithms. For example, Yasen et al. [31] developed an Extreme Learning Machine (ELM) model to predict the strength of cellular concrete, achieving greater accuracy than Multivariate Adaptive Regression Splines (MARS), M5 Tree, and SVR, demonstrating its usefulness for improving quality control and reducing physical testing. Similarly, Asteris et al. [32] introduced a hybrid learning model (Hybrid Ensembling of Surrogate Machine Learning Models, HENSM) that integrates ANNs, MARS-L/C (linear (L) and cubic (C) variants of the Multivariate Adaptive Regression Splines), Gaussian Process Regression (GPR), and Minimax Probability Machine Regression (MPMR) within a neural network, achieving more accurate and stable predictions with lower risk of overfitting and potential implications for the design of more sustainable concretes. Other studies, such as that by Kandiri et al. [33], combined ANNs with optimization algorithms (Salp Swarm Algorithm (SSA), Genetic Algorithm (GA), Grasshopper Optimization Algorithm (GOA)) to predict the strength of concrete with recycled aggregates, highlighting the robustness of hybrid approaches. Likewise, Bui et al. [34] applied an ANN optimized with the Whale Optimization Algorithm (WOA), outperforming other evolutionary methods (Dragonfly Algorithm (DA) and Ant Colony Optimization (ACO)). Omran et al. [35] compared nine data mining models for concretes with mineral admixed and found that GPR offered the highest predictive accuracy, especially when combined with ensemble methods. Alshihri et al. [36] also demonstrated the effectiveness of Cascade Correlation Neural Networks (CCNN) in predicting the strength of lightweight concrete at different curing ages, significantly reducing testing costs and time. Beyond numerical prediction, several studies have employed AI models to classify concretes according to their compressive strength. Alghamdi [37] used Decision Tree methods to accurately categorize high-strength mix designs. Zhao [38], applying the Naïve Bayes model, reliably identified strength categories in ultra-high-performance concretes, evidencing its usefulness for quality control. Models such as Random Forest (RF) [39,40] have proven robust against noisy data, while Decision Trees, SVM, and k-NN have shown variable results depending on the mix design and data distribution [41,42].

In addition to these advances, recent research has increasingly adopted more powerful machine learning frameworks to model the behavior of sustainable concretes. For example, Khan et al. [43] employed Extreme Gradient Boosting (XGBoost) to predict the mechanical performance of waste-incorporated concretes, demonstrating high accuracy and robust feature sensitivity analysis. Zhang et al. [44] introduced a hybrid RF–GWO (Grey Wolf Optimizer)–XGBoost model for geopolymer blends, highlighting the potential of ensemble techniques in sustainability-oriented applications. Other developments include the work of Demirtürk [45], who optimized XGBoost and Light Gradient Boosting Machine (LightGBM) for high-performance concretes, and Fei et al. [46], who applied ensemble ML models, including XGBoost, Random Forest, LightGBM, and Adaptive Boosting (AdaBoost), to predict the compressive strength of recycled powder mortar.

Most previous studies have focused on predicting the compressive strength of conventional or recycled concrete. In contrast, very few studies have developed machine learning classification methods for eco-friendly concretes produced with glass powder as a partial cement substitute. Therefore, in this study, we focus on analyzing different ML algorithms to classify these concretes based on their compressive strength. The experimental dataset used in this study was specifically generated with systematic variations in glass powder content, allowing for a detailed analysis of its influence on strength classes. The goal of this research is to develop and identify the most reliable and generalizable methods for quality control and mix optimization, thereby reducing the need for destructive testing and the associated material and time costs.

## 2. Materials and Methods

For this study, data were collected at the materials laboratory of the School of Engineering in the Universidad Politécnica de Madrid between 2004 and 2019. During this period, 846 concrete specimens with varying compositions and mix designs were prepared.

The first step of the study involved the characterization of the materials used in concrete production to ensure their quality and the representativeness of the experimental tests.

### 2.1. Material Characterization

#### 2.1.1. Glass Powder

The glass powder used in this study was obtained from two sources: ceramic industry waste (frits) and end-of-life recycled glass containers and packaging, which, due to their properties, cannot be reintroduced into the glass manufacturing process and are disposed of in inert landfills.

The glass powders were characterized using three particle size parameters: d_10_, d_50_, and d_90_, which represent the particle diameters below which 10%, 50%, and 90% of the sample particles, respectively, are smaller than the indicated size. For the purpose of this study, only the d_50_ value is used to characterize the different batches of ground glass. All analyses were performed using a COULTER LS 100Q laser granulometer (Beckman Coulter, Inc., Brea, CA, USA), maintaining a cell obscuration coefficient between 8% and 12%.

Three batches of glass powder were selected to evaluate the influence of particle size on the compressive strength of the concrete specimens, corresponding to d_50_ = 33 ± 1 µm, 16 ± 1 µm, and 11 ± 1 µm. A summary of the results is presented in Table 1. A Retsch PM 400 planetary ball mill (Retsch GmbH, Haan, Germany) was used to grind the glass to a particle size of approximately 16 µm.

#### 2.1.2. Cement

A commercial Portland cement type CEM I 52.5 R (Cementos Portland Valderrivas, Morata de Tajuña, Madrid, Spain) was used in this study. The cement exhibited a density of 3.12 g/cm^3^, a specific surface area of 4440 cm^2^/g, and a greenish-gray color. The particle size distribution analysis indicated that 41.5% of the total volume consisted of particles smaller than 8 μm, while 99.7% were below 96 μm.

The chemical composition of the CEM I 52.5 R cement, expressed as mass percentage, was as follows: CaO (65%), SiO_2_ (19%), Al_2_O_3_ (5.5%), Fe_2_O_3_ (2.65%), SO_3_ (2.0%), MgO (2.0%), Na_2_O (0.15%), and K_2_O (0.7%).

#### 2.1.3. Aggregates

The aggregates used were of siliceous nature and non-reactive. A fine aggregate (sand) with a particle size below 4 mm was employed, together with coarse aggregates in two size ranges: 4–12 mm and 12–20 mm.

### 2.2. Preparation of Samples

To evaluate the different properties of the concrete, a series of specimens were prepared in accordance with the UNE-EN 12390-2 standard [47]. Each batch featured a distinct composition of the components constituting the mixture.

All mixtures were thoroughly homogenized and cast into cylindrical molds measuring 10 × 30 cm. The specimens were then compacted, demolded after 24 h, and cured in a humidity-controlled chamber at 20 °C for 1, 3, 7, 14, 28, 56, 90, 180, 270, and 360 days. After the curing period, compressive strength tests were carried out in accordance with UNE 83-304-84 [48] to determine the compressive strength of the concrete.

#### Summary of Mix Compositions Used in the Tested Specimens

Table 2 presents a summary of the mix compositions used in the preparation of the 846 specimens tested for compressive strength throughout the study period.

### 2.3. Mechanical Properties of Concrete

After the specimens reached the specified curing ages (1, 3, 7, 14, 28, 56, 90, 180, 270, and 360 days), compressive strength tests were conducted to determine the mechanical performance of the concrete. The results for each specimen were recorded according to its mixture composition and curing time. Table 3 summarizes the compressive strength values obtained as a function of the curing time.

The concrete samples were categorized into four strength classes: non-structural, ordinary, high, and very high, according to Eurocode 2 (EN 1992-1-1) [15]. Specifically, the non-structural category corresponds to compressive strength < 20 MPa, ordinary to 20–40 MPa, high to 40–60 MPa, and very high to >60 MPa. This standardized classification framework was used as the target variable for the machine learning models, enabling the systematic evaluation of the algorithms’ performance and facilitating comparisons across eco-friendly concretes incorporating glass powder.

### 2.4. Data Processing and Methodology of Machine Learning Models

**Figure 1:**
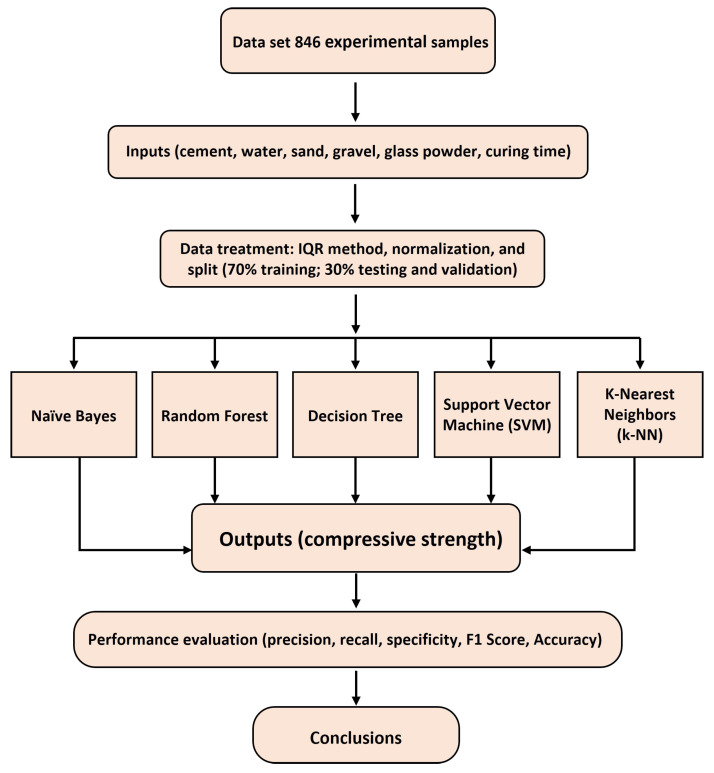
General flow chart of the machine learning methodology used for accurate classification of compressive strength in eco-friendly concrete.

To assess the predictive performance of machine learning algorithms in classifying the compressive strength of eco-friendly concrete, a systematic data processing and modeling procedure was implemented. The dataset was preprocessed by detecting outliers using the interquartile range (IQR) method, removing measurement errors, and preserving valid extreme values. Numerical features (cement, water, fine and coarse aggregates, glass powder, and curing age) were normalized to the range [0,1] using Min–Max scaling, and categorical variables were one-hot encoded. Subsequently, the dataset was split into training (70%), testing and validation (30%) subsets, with stratified sampling to preserve the distribution of strength classes. Figure 1 illustrates the overall workflow of the methodology, summarizing the main stages of data collection, processing, model selection, and verification.

#### Description of the Models

Several ML classification models were evaluated, including Support Vector Machines (SVM), Naïve Bayes, Random Forest, Decision Trees, and k-Nearest Neighbors (k-NN). All models were implemented using the open-source KNIME Analytics Platform (version 5.5) [49], which is particularly suitable for data analysis due to its user-friendly interface and low computational requirements.

The Naïve Bayes model is a supervised ML algorithm used for classification tasks [50], that is, to predict the category or class to which a given observation belongs. This algorithm is based on Bayes’ Theorem and assumes that the features describing an instance are conditionally independent, given the class label. Although this assumption rarely holds true in real-world data, the model remains highly effective and robust across various applications. In this study, the model was trained using 70% of the total dataset, with the remaining 30% reserved for validation. This model predicts strength assuming conditional independence between the input features. Probabilistic distributions are estimated for each feature (cement, water, aggregates, glass powder) relative to discrete output categories or ranges of compressive strength, and predictions are obtained by combining the probabilities according to Bayes’ theorem. The default implementation assumed a Gaussian distribution for numerical features, and hyperparameter tuning was not required due to the model’s simplicity.

Random Forest is a supervised ensemble learning algorithm used for both classification and regression tasks. Its main objective is to predict the class of a given instance by combining the outputs of multiple Decision Trees, to achieve higher accuracy and robustness [51]. In this study, 70% of the total dataset was used for model training, and 30% was reserved for validation. The algorithm constructs an ensemble of decision trees, each trained on random subsets of the input features to generate splitting rules based on cement, water, aggregates, and glass powder. This design allows the model to capture complex nonlinear interactions between the mixed components and reduces overfitting, thereby improving generalization. Once trained, the Random Forest produces its final prediction by aggregating the outputs of all individual trees and assigning each concrete specimen to the class receiving the most votes. The model was configured with 100 trees, a maximum depth of 10, and a minimum sample split of 2. Hyperparameters were optimized using a grid search with 5-fold cross-validation to maximize classification accuracy.

The Decision Tree algorithm is a supervised learning model widely used for classification, as it hierarchically segments data according to the most relevant features [52]. In this work, the model predicts compressive strength classes by recursively splitting the dataset based on thresholds of input features (cement, water, aggregates, glass powder, curing age). The tree structure captures hierarchical relationships among variables and provides class predictions at the leaf nodes. The algorithm was trained with 70% of the dataset, while the remaining 30% was used for validation. At each node, the tree selected the attribute that maximized class separation, effectively dividing the data into increasingly homogeneous subgroups. This approach revealed clear relationships between mixture components, curing time, and compressive strength class, showing that specific composition ranges consistently influenced mechanical performance. After training, the model assigned each new specimen to the class corresponding to the terminal (leaf) node it fell into. The interpretable tree structure also made it possible to visualize the key factors governing compressive strength. A configuration with a maximum tree depth of 8 and a minimum of one sample per leaf, without pruning, was adopted based on validation set performance.

The k-Nearest Neighbors (k-NN) algorithm is an instance-based supervised learning method used for classification tasks [53]. In this study, it was applied to predict the compressive strength class of concrete specimens based on their mix composition (cement, water, aggregates, and glass powder) and curing time. The algorithm measures the distance between a new sample and all samples in the training set, identifies the k most similar mixtures using the Euclidean distance metric, and assigns the new specimen to the most frequent class among these neighbors. This allows the model to capture local relationships in the dataset, where mixtures with similar compositions tend to exhibit similar compressive strength classifications. The model was trained using 70% of the dataset, with the remaining 30% employed for validation. The number of neighbors was set to k = 5, selected based on cross-validation performance.

The Support Vector Machine (SVM) is a supervised ML algorithm widely used for regression and classification tasks due to its robustness and ability to generalize to unseen data [54]. In this study, SVM was employed to model the compressive strength of eco-friendly concretes. The input variables (cement, water, aggregates, and glass powder) were used to capture nonlinear relationships through the RBF kernel. SVM constructs an optimal hyperplane in a high-dimensional feature space, with support vectors defining the regression function based on the most influential samples. When a new mixture is provided, the model evaluates its position relative to this hyperplane to predict compressive strength. As with the other models, 70% of the data were used for training and 30% for validation. Key hyperparameters were tuned using grid search with 10-fold cross-validation (RBF kernel, C = 10, gamma = 0.1) to ensure reliable predictive performance.

**Figure 2:**
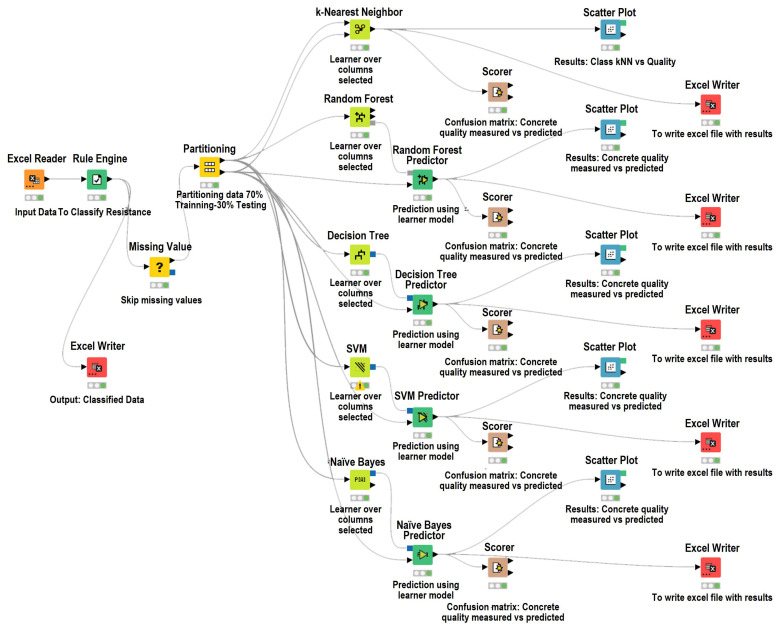
Overview of the KNIME-based workflow used for the development, training, and evaluation of the machine learning models for accurate classification of compressive strength in eco-friendly concrete.

As a summary, Figure 2 illustrates the operational framework of the machine learning models developed using the KNIME Analytics Platform (version 5.5), providing a structured representation of the workflow implemented in each case.

## 3. Results

Table 4 shows the results obtained by laboratory-measured compressive strength of eco-friendly cement samples with the classifications produced by the ML models. This representation highlights the models’ capability to categorize the cements according to their compressive performance across different curing times.

A comprehensive evaluation of the obtained results will be conducted using robust performance metrics designed to rigorously quantify the predictive accuracy and reliability of the proposed artificial intelligence models for the classification of eco-friendly concrete mixtures based on their compressive strength.

### Model Performance Evaluation

To assess the predictive capability and robustness of the proposed ML models, several performance metrics were employed. All metrics were derived from the confusion matrix presented in Table 5, which summarizes the classification outcomes for the validation dataset. These metrics quantify the models’ ability to accurately classify the compressive strength classes of concrete specimens based on their mix composition and curing time. Standard statistical indicators, including accuracy, precision, recall, and F1-score, were calculated to provide a comprehensive understanding of both the overall and class-specific performance of each algorithm.

Table 6 presents the performance metrics derived from the confusion matrix, used to evaluate the ML models applied in this study. Accuracy measures the proportion of correct predictions over the total number of instances, while precision and recall provide a more detailed, class-specific analysis: precision indicates how reliable the positive predictions are, and recall reflects the model’s ability to identify all actual instances of a given class. The F1-score, as the harmonic mean of precision and recall, provides a balanced measure of overall performance. Finally, specificity complements recall by assessing the model’s ability to avoid false positives. Together, these metrics allow for a comprehensive comparison of the models, considering both their global performance and their capacity to correctly distinguish between different concrete types.

Table 7 complements the information provided in the previous paragraph by presenting the actual values of these metrics for each ML model applied to the concrete dataset.

A detailed examination of the model performance metrics highlights significant differences in predictive behavior across the evaluated algorithms. Naïve Bayes consistently achieved high values across all metrics for all strength categories (precision and recall > 0.85; F1-score ~0.95; overall accuracy 0.95), demonstrating both excellent positive case identification and strong resistance to false positives. This balance indicates a robust capacity to generalize across heterogeneous data, making it the most reliable model for comprehensive classification.

Random Forest exhibited generally strong performance, with moderate to high precision and recall (accuracy 0.76), yet a noticeable decline in recall for the minority classes (Non-structural and Very high-strength) suggests some sensitivity to class imbalance. Its specificity remained high, indicating correct identification of negative cases even when positive instances were misclassified.

Decision Tree achieved perfect metrics (1.0) across all categories, reflecting overfitting rather than true predictive capability. While this ensures flawless performance on the training set, the lack of generalization limits its utility for unseen data.

SVM and k-NN displayed highly uneven metrics. SVM attained perfect precision and recall only in extreme classes (Non-structural and Very high-strength), with poor recall in intermediate categories (e.g., 0.10 for Ordinary structural), indicating failure to capture subtle inter-class boundaries. Similarly, k-NN performed acceptably for low-strength concrete but almost completely failed in High-strength classes (precision 0.01) and very high-strength (recall 0.10), reflecting strong sensitivity to local data distribution and feature scaling.

In conclusion, the comparison of machine learning models highlights their respective strengths and limitations in predicting concrete strength classes. Naïve Bayes consistently achieved high accuracy across all classes, demonstrating robust generalization. Random Forest performed well for “Ordinary structural” and “High strength” classes but showed lower recall for minority classes. SVM excelled in “Non-structural” and “Very high-strength” classes but was less effective for intermediate classes, while k-NN performed well for “Ordinary structural” but struggled with higher strength classes due to sensitivity to local patterns. Decision Tree achieved perfect classification, likely reflecting overfitting. Overall, these results illustrate the analytical focus of each model. SVM emphasizes margin-based separation, Naïve Bayes leverages probabilistic feature relationships, Random Forest and Decision Tree use hierarchical splits, and k-NN relies on local similarity, providing clear guidance for selecting the most appropriate method based on the target concrete class and dataset characteristics.

**Figure 3:**
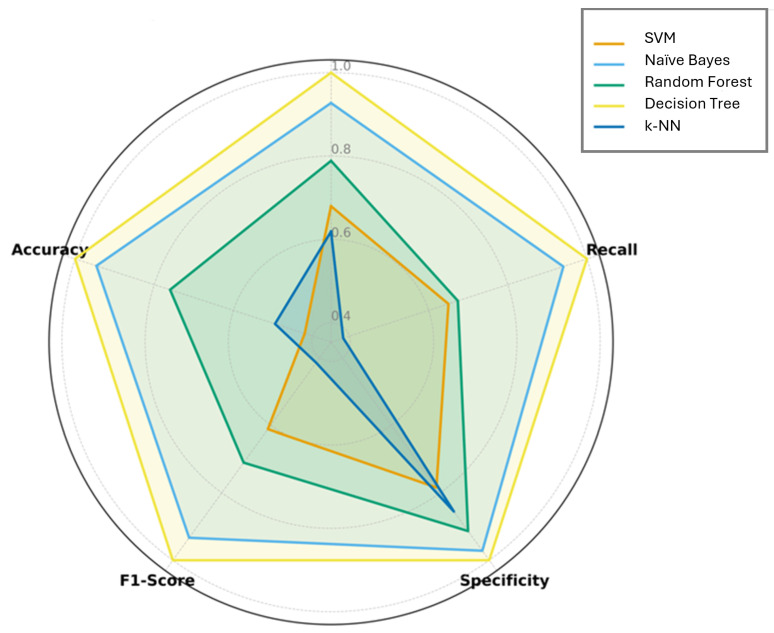
Radar chart comparison of performance metrics of the Machine Learning models for eco-friendly concrete classification according to its compressive strength.

Figure 3 illustrates a radar chart comparing the global performance of the evaluated machine learning models across five metrics: Precision, Recall, Specificity, F1-Score, and Accuracy.

## 4. Discussion

The comparative evaluation of five machine learning algorithms—Naïve Bayes, Random Forest, Decision Tree, Support Vector Machine (SVM), and k-Nearest Neighbors (k-NN), for classifying the compressive strength of eco-friendly concretes incorporating recycled glass powder has revealed substantial differences in predictive behavior and practical implications for sustainable material design. These findings not only highlight the computational performance of each model but also provide valuable insight into their suitability for guiding the development and structural use of environmentally responsible cementitious composites.

The Naïve Bayes model exhibited the highest overall performance (accuracy ≈ 0.95), maintaining balanced precision and recall across all strength categories. Its strong predictive capability for high and ultra-high strength concretes aligns with prior studies by Zhao et al. [38], which attribute this robustness to the algorithm’s probabilistic structure and its ability to effectively handle datasets with moderate collinearity and clearly separated quantitative features. In the context of glass powder modified concretes, this performance suggests that the material’s mechanical behavior remains sufficiently distinct across strength classes to be probabilistically separable, supporting the potential use of Naïve Bayes as an efficient, low-complexity model for early-stage material screening.

The Random Forest algorithm achieved comparably strong results (accuracy ≈ 0.76), especially in the ordinary structural and high-strength categories. Its ensemble-based architecture, which aggregates multiple decision trees through bagging, allows it to capture nonlinear interactions between material components and curing parameters. These results corroborate findings by Omran et al. [35] and Asteris et al. [32], who reported similar advantages of tree-based models for predicting concrete mechanical properties. In this study, Random Forest demonstrated high robustness to noise and variability arising from the inclusion of glass powder, confirming its capability for accurate classification and potential real-time monitoring of eco-concretes during production and curing. However, the slight underperformance in the very high-strength category suggests the need for further class balancing or feature engineering when dealing with minority strength ranges.

Conversely, the Decision Tree model achieved perfect performance (accuracy = 1.00), a result indicative of overfitting rather than true generalization. Although this behavior superficially suggests exceptional precision, it compromises reliability when predicting unseen data. Similar overfitting tendencies have been reported by Asteris et al. [32], who noted that decision tree–based models tend to overfit when the number of predictor variables is high or when class distinctions are subtle. Nonetheless, the interpretability and transparent decision rules of this model make it a valuable analytical tool for exploring parameter–response relationships, particularly for visualizing the influence of glass powder content, water-to-binder ratio, or curing age on strength development. In practice, Decision Trees may serve as an explanatory component within hybrid or ensemble frameworks, enhancing interpretability without sacrificing predictive accuracy.

The SVM model, in contrast, exhibited inconsistent performance (accuracy ≈ 0.42), performing well only in the extreme categories (non-structural and ultra-high strength). This pattern mirrors findings from previous studies [41], where the margin-based nature of support vector machines is highly effective for well-separated classes but limited in cases of overlapping or complex nonlinear relationships unless kernel parameters are carefully optimized. Given the compositional heterogeneity of eco-friendly concretes, where particle morphology, pozzolanic activity, and curing kinetics interact non-linearly, the limited adaptability of the SVM underscores the necessity of kernel tuning or hybridization for improved predictive stability in sustainable material systems.

Similarly, the k-Nearest Neighbors (k-NN) model demonstrated the lowest overall accuracy (≈0.49) and weak generalization in the high and very high-strength categories. Although it performed reasonably well for ordinary concrete, its dependence on local data density and sensitivity to feature scaling reduced its robustness in heterogeneous datasets. These limitations, reported also by Kandiri et al. [33], highlight that k-NN is more suited for qualitative or semi-quantitative classification tasks, such as preliminary mixture grouping or process monitoring, rather than for rigorous strength classification.

In summary, the comparative performance trends are consistent with the evidence obtained from the feature importance analysis. Naïve Bayes and Random Forest outperformed SVM, k-NN, and Decision Tree. Naïve Bayes benefited from well-differentiated feature distributions, and Random Forest leveraged its ensemble architecture to capture nonlinear interactions. The feature importance analysis confirmed that cement content, glass powder ratio, and curing age are the dominant variables influencing strength classification, while aggregate ratios and water content play a secondary role. These findings not only explain the superior accuracy of Naïve Bayes and Random Forest but also highlight the practical value of feature-based information for optimizing the design of eco-friendly concrete mixes.

From a broader perspective, these results emphasize that probabilistic and ensemble-learning methods, such as Naïve Bayes and Random Forest, provide the most balanced combination of accuracy, generalization, and interpretability for classifying eco-friendly concretes. Their strong predictive performance confirms that the incorporation of recycled glass powder as a partial cement replacement does not introduce excessive data noise or instability, thereby reinforcing its potential as a viable and sustainable substitute for structural applications. Furthermore, the consistent increase in predicted strength classification with curing age across all models reflects the expected hydration and pozzolanic activity trends of blended cements, validating both the experimental observations and the physical relevance of the models.

In contrast, while the Decision Tree model excels in interpretability, its tendency to overfit limits its predictive generalization, making it more suitable for exploratory or explanatory analyses. Similarly, SVM and k-NN demonstrate effectiveness primarily in extreme strength categories, where class boundaries are more distinct. Collectively, these outcomes are consistent with trends reported in recent literature [31,32,33,34,35,36], which indicate that probabilistic and ensemble-based approaches consistently outperform deterministic and proximity-based models in the classification of materials characterized by high compositional variability.

The discrepancies observed among the algorithms can be partially explained by the intrinsic differences between glass powder concrete and conventional mixes. The incorporation of glass powder as a partial cement replacement results in a more eco-friendly concrete, which exhibits a distinctive characteristic compared to conventional concretes: it prolongs the setting reactions due to the extension of its pozzolanic reactions [54]. This modification affects the hydration kinetics and microstructure, producing denser matrices, but also greater variability in early-age strength due to the pozzolanic reaction. This heterogeneity can influence the predictive behavior models, particularly in algorithms sensitive to data distribution, such as SVM and k-NN. In contrast, ensemble and tree-based methods better capture nonlinear dependencies and compositional interactions, which explain their greater accuracy and recall in most strength classes. However, despite these potential sources of variability, the experimental data did not show significant instability in the compressive strength measurements. This can be attributed to the small particle size of the recycled glass powder, which promotes uniform dispersion; the pozzolanic reaction, which provides additional C–S–H gel without generating abrupt strength fluctuations; and the low substitution levels (1–2% by volume), which preserve the homogeneity of the mixture and prevent clustering or segregation. Together, these factors support the reliability and consistency of the dataset used for machine learning classification.

## 5. Conclusions

The results of this study demonstrate that ML models constitute effective and sustainable tools for classifying the compressive strength of eco-friendly concretes in which a fraction of the cement is replaced with glass powder. This data-driven approach contributes to more efficient resource management, supports the principles of the circular economy, and reduces the need for extensive destructive testing. Based on model performance, the key findings can be summarized as follows:

- The Naïve Bayes classifier achieved the highest overall accuracy (≈0.95) demonstrating a balance between accuracy and comprehensiveness across all strength categories. Random Forest also performed reliably (accuracy ≈ 0.76), effectively capturing the nonlinear interactions between mixture components and curing parameters. Its consistent performance confirms that partial cement replacement with glass powder does not introduce significant instability into the data, reinforcing its potential as a viable and sustainable substitute for structural concrete applications.
- The Decision Tree model achieved perfect accuracy (1.00), indicating possible overfitting; Nevertheless, its interpretability makes it particularly valuable for explanatory analysis and understanding the relative influence of mixture parameters such as glass powder content, water-to-binder ratio, and curing age on concrete strength.
- The SVM and k-NN methods, with accuracies of 0.42 and 0.50, respectively, demonstrated competitive performance in extreme strength categories but lower overall accuracy in heterogeneous datasets, limiting their applicability for generalized prediction. However, their simplicity and responsiveness to local patterns make them suitable for preliminary mixture screening, process monitoring, or rapid on-site quality control.

Overall, the findings highlight that probabilistic and ensemble-based methods outperform deterministic and proximity-based algorithms when classifying materials with high compositional variability. The choice of model should therefore be guided by the specific objectives of the study, balancing interpretability, accuracy, and generalization depending on the intended application.

In summary, AI-based models, such as Naïve Bayes and Random Forest, can optimized mix design, enable real-time monitoring during production, and contribute to non-destructive testing by providing early strength class predictions. This scalable framework improves decision-making, efficiency, and reliability in sustainable construction and can accelerate the adoption of low-carbon materials and the efficient use of resources, supporting the broader goals of sustainable construction and the circular economy in the cement and concrete industry.

A potential future line of research could explore several avenues to further strengthen and expand the findings of this study. For example, studying other types of additives to cement to produce different kinds of eco-friendly concretes (such as ground granulated blast furnace slag, fly ash, eggshell, etc.) could enhance the model’s applicability. Conducting external validation using an independent dataset, processed through the same preprocessing steps but without retraining the model, could also help assess performance across diverse contexts. Moreover, examining and applying data balancing techniques for categories with fewer samples may contribute to improved generalizability and robustness of the model.

## Figures and Tables

**Table 1:** Granulometric characterization of glass powder according to milling time.

Glass Powder	Grinding Time (h)	d_10_	d_50_	d_90_
1	2.5	2.92±0.01	33±1	110±2
2	4.25	1.96±0.01	16±1	59±2
3	5	1.65±0.01	11±1	43±2

**Table 2:** Summary of the composition of the tested specimens (kg/m^3^).

Constituent	Minimum	Maximum	Mean	Standard Deviation
Cement	78	595	347	79
Water	102	266	178	23
Gravel	649	1355	1146	128
Sand	20	980	675	158
Glass powder	0	941	96	128

**Table 3:** Compressive strength (MPa) results of eco-friendly concrete samples versus curing time.

Curing Time (Days)	Minimum	Maximum	Mean	SD	N
365	25.72	76.25	50.29	13.17	39
270	44.79	50.09	47.00	2.59	15
180	10.37	74.45	45.89	14.65	55
90	8.67	95.76	46.66	16.97	105
56	11.12	55.40	35.72	9.75	119
28	0.82	81.41	36.52	12.59	216
14	7.73	44.11	28.18	10.98	39
7	1.54	69.72	29.12	11.37	201
3	0.51	33.38	18.22	9.87	40
1	2.46	29.82	12.12	8.54	17

**Table 4:** Results obtained in the classification of eco-friendly concrete samples, based on compressive strength and curing time, measured in the laboratory and predicted by the five machine learning models analyzed.

Curing Time	1	3	7	14	28	56	90	180	270	365
Measured compressive strength										
Ordinary structural	7	7	12	1	6	1	2	1	0	0
Non-structural	2	7	56	4	53	26	10	4	0	5
High-strength	0	0	9	3	42	9	23	8	1	7
Very high-strength	0	0	7	0	4	0	8	2	0	4
Predicted by the SVM model										
Ordinary structural	7	6	7	1	4	0	2	1	0	0
Non-structural	0	0	2	5	9	4	12	4	0	4
High-strength	2	8	68	2	78	32	20	8	1	8
Very high-strength	0	0	3	0	4	0	8	2	0	4
Predicted by the Naïve Bayes model										
Ordinary structural	9	14	80	8	92	39	43	15	1	16
Non-structural	1	7	58	14	52	26	9	4	0	5
High-strength	0	0	9	2	28	9	24	8	1	7
Very high-strength	0	0	3	0	6	0	8	2	0	4
Predicted by the Random Forest model										
Ordinary structural	7	7	11	1	6	1	2	1	0	0
Non-structural	2	7	57	4	53	26	10	4	0	5
High-strength	0	0	9	3	29	9	23	8	1	7
Very high-strength	0	0	3	0	4	0	8	2	0	4
Predicted by the Decision Tree model										
Ordinary structural	7	7	12	1	6	1	2	1	0	0
Non-structural	2	7	56	4	53	26	10	4	0	5
High-strength	0	0	9	3	29	9	23	8	1	7
Very high-strength	0	0	3	0	4	0	8	2	0	4
Predicted by the k-Nearest Neighbors model										
Ordinary structural	3	3	7	1	6	1	5	0	0	0
Non-structural	3	10	51	5	60	25	11	5	0	2
High-strength	3	1	19	2	24	10	23	9	1	14
Very high-strength	0	0	3	0	2	0	4	1	0	0

**Table 5:** Confusion matrices showing the classification performance of the machine learning models.

Model	Concrete Classification Based on Compressive Strength				
		Ordinary Structural	Non-Structural	High-Strength	Very High-Strength
SMV	Ordinary structural	17	0	150	0
	Non-structural	0	28	9	0
	High-strength	23	0	66	0
	Very high-strength	0	0	0	21
Naïve Bayes	Ordinary structural	159	4	4	0
	Non-structural	5	32	0	0
	High-strength	23	0	66	0
	Very high-strength	0	0	0	21
Random Forest	Ordinary structural	137	6	24	0
	Non-structural	16	20	1	0
	High-strength	15	0	73	1
	Very high-strength	4	0	8	9
Decision Tree	Ordinary structural	167	0	0	0
	Non-structural	0	37	0	0
	High-strength	0	0	89	0
	Very high-strength	0	0	0	21
k-NN	Ordinary structural	137	6	24	0
	Non-structural	16	20	0	0
	High-strength	15	0	1	1
	Very high-strength	4	0	73	9

**Table 6:** Performance metrics and their mathematical formulas.

Metric	Mathematical Formula
Precision	$P=\frac{TP}{TP+FP}$
Recall	$R=\frac{TP}{TP+FN}$
Specificity	$S=\frac{TN}{TN+FP}$
F1-Score	$F1=\frac{2*\left(P*R\right)}{P+R}$
Accuracy	$A=\frac{TP+TN}{TP+TN+FP+FN}$

Where: TP: True Positives; TN: True Negatives; FP: False Positives; FN: False Negatives.

**Table 7:** Results obtained for the performance metrics of the machine learning models studied in the classification of eco-friendly concrete based on its compressive strength.

Model	Metrics					
		Precision	Recall	Specificity	F1-Score	Accuracy
SMV	Ordinary structural	0.42 ± 0.03	0.10 ± 0.03	0.84 ± 0.02	0.16 ± 0.02	0.42 ± 0.04
	Non-structural	1.00 ± 0.00	0.76 ± 0.05	1.00 ± 0.00	0.86 ± 0.03	
	High-strength	0.29 ± 0.04	0.74 ± 0.05	0.29 ± 0.03	0.42 ± 0.04	
	Very high-strength	1.00 ± 0.00	1.00 ± 0.00	1.00 ± 0.00	1.00 ± 0.00	
Naïve Bayes	Ordinary structural	0.95 ± 0.01	0.95 ± 0.01	0.94 ± 0.01	0.95 ± 0.01	0.95 ± 0.01
	Non-structural	0.89 ± 0.02	0.86 ± 0.03	0.99 ± 0.01	0.88 ± 0.02	
	High-strength	0.96 ± 0.01	0.94 ± 0.02	0.96 ± 0.01	0.95 ± 0.01	
	Very high-strength	0.91 ± 0.02	1.00 ± 0.00	1.00 ± 0.02	0.96 ± 0.01	
Random Forest	Ordinary structural	0.80 ± 0.02	0.82 ± 0.03	0.87 ± 0.02	0.81 ± 0.02	0.76 ± 0.02
	Non-structural	0.77 ± 0.03	0.56 ± 0.05	0.96 ± 0.02	0.64 ± 0.04	
	High-strength	0.69 ± 0.03	0.82 ± 0.03	0.85 ± 0.03	0.75 ± 0.03	
	Very high-strength	0.90 ± 0.02	0.50 ± 0.04	0.98 ± 0.02	0.64 ± 0.03	
Decision tree	Ordinary structural	1.00 ± 0.00	1.00 ± 0.00	1.00 ± 0.00	1.00 ± 0.00	1.00 ± 0.00
	Non-structural	1.00 ± 0.00	1.00 ± 0.00	1.00 ± 0.00	1.00 ± 0.00	
	High-strength	1.00 ± 0.00	1.00 ± 0.00	1.00 ± 0.00	1.00 ± 0.00	
	Very high-strength	1.00 ± 0.00	1.00 ± 0.00	1.00 ± 0.00	1.00 ± 0.00	
k-NN	Ordinary structural	0.80 ± 0.02	0.82 ± 0.03	0.78 ± 0.02	0.81 ± 0.02	0.50 ± 0.03
	Non-structural	0.77 ± 0.03	0.56 ± 0.02	0.98 ± 0.02	0.64 ± 0.04	
	High-strength	0.01 ± 0.01	0.51 ± 0.02	0.67 ± 0.03	0.18 ± 0.01	
	Very high-strength	0.90 ± 0.02	0.10 ± 0.03	1.00 ± 0.01	0.19 ± 0.02	

## Data Availability

The original contributions presented in this study are included in the article. Further inquiries can be directed to the corresponding author.
